# Granulocyte colony-stimulating factor combined with SOFA score for mortality prediction in patients with sepsis

**DOI:** 10.1097/MD.0000000000040926

**Published:** 2024-12-27

**Authors:** Xiaomeng Fu, Ye Zhang, Junyu Wang, Yugeng Liu, Bing Wei

**Affiliations:** a Department of Infectious Disease and Clinical Microbiology, Beijing Chao-Yang Hospital, Capital Medical University, Beijing, China; b Emergency Medicine Clinical Research Center, Beijing Chaoyang Hospital, Capital Medical University, Beijing, China; c Beijing Key Laboratory of Cardiopulmonary Cerebral Resuscitation, Clinical Center for Medicine in Acute Infection, Capital Medical University, Beijing, China.

**Keywords:** APACHE II, G-CSF, granulocyte colony-stimulating factor, sepsis, SOFA

## Abstract

**Background:**

Sepsis in emergency departments is a prevalent occurrence characterized by high hospitalization rate and mortality. The granulocyte colony-stimulating factor (G-CSF) is an indicator for identifying patients with sepsis.

**Methods:**

A total of 171 patients with sepsis were included in our study who were admitted to the emergency department of Beijing Chaoyang Hospital affiliated with Capital Medical University from October 2020 to April 2021. Out of these patients, 122 did not survive on day 28. Laboratory tests, the sequential organ failure assessment (SOFA) score and the acute physiology and chronic health evaluation II (APACHE II) were calculated. Logistic regression and receiver operating characteristic curve were used to analyze the predictive value of G-CSF for 28-day mortality patients with sepsis.

**Results:**

There were significant differences in G-CSF, SOFA, APACHE II, systolic blood pressure (SBP), mean arterial pressure, lactate, and albumin between the survivor and non-survivor groups (*P* < .05). The multivariate regression analysis showed that G-CSF, SOFA, APACHE II, and SBP were independent risk factors for 28-day mortality in patients with sepsis. There was no comparative with significant differences in receiver operating characteristic curves of G-CSF, SOFA, and APACHE II for 28-day mortality in patients with sepsis (*Z*_1_ = 1.381, *P* = .167; *Z*_2_ = 0.095, *P* = .924).

**Conclusions:**

The G-CSF, SOFA, APACHE II, and SBP were identified as independent risk factors for mortality among patients with sepsis. Particularly, G-CSF and SOFA exhibited a high level of predictability for 28-day mortality in this population.

## 1. Introduction

Sepsis is defined as a life-threatening organ dysfunction induced by a dysregulated host response to infection.^[1]^ It is a prevalent occurrence in emergency departments, whereby a substantial number of patients with sepsis tend to suffer from severe morbidity or mortality despite being subjected to intensive care. Therefore, the effective prognostic evaluation of sepsis patients is significant. Various prognostic indicators have been proposed, and several scoring systems have been externally validated and globally used to calculate the severity of organ dysfunction.^[2–5]^ The use of sequential organ failure assessment (SOFA) and acute physiology and chronic health evaluation II (APACHE II) scores to assess changes in the status of patients over time in the intensive care unit has been validated to represent better mortality prediction.^[6]^ Recently, several biomarkers, including procalcitonin, C-reactive protein (CRP), lactate (LAC), and interleukin (IL)-6, have helped evaluate the severity and prognosis of patients with sepsis.^[7–10]^ However, none of these indicators have 100% sensitivity or 100% specificity. G-CSF, which is an acronym for granulocyte colony-stimulating factor, is a multifunctional cytokine that is synthesized by various immune cells, including macrophages and endothelial cells. This G-CSF is responsible for stimulating the bone marrow to produce granulocytes and stem cells, which are subsequently released into the bloodstream. Additionally, G-CSF is also referred to by several other names, including colony-stimulating factor 3, CSF3, C17orf33, and CSF3OS.^[11]^ It exhibits properties of a cytokine and hormone and stimulates a range of effects, including promoting survival, proliferation, differentiation, and the function of neutrophil precursors and mature neutrophils. Thus, the level of G-CSF might be indicative of the prognosis of patients with sepsis. However, there are very few studies on the correlation between G-CSF and the prognosis of patients with sepsis. Consequently, the aim of this investigation is to assess the prognostic significance of G-CSF in patients with sepsis.

## 2. Patients and methods

### 2.1. Patients

Typically, 171 patients with sepsis were included who were admitted to the emergency department of Beijing Chaoyang Hospital, affiliated with Capital Medical University, from October 2020 to April 2021. The inclusion criteria for the research encompassed sepsis patients and aged ≥18. Sepsis severity defined as when SOFA score was ≥2 points according to the Sepsis-3 classification criteria.^[1]^ Conversely, the exclusion criteria comprised individuals who were pregnant, had malignant tumors, hematological diseases, or connective tissue disease. The patients were divided into survivor group and non-survivor group according to their survival status at the 28-day follow-up. The study was approved by the Ethics Committee of the Beijing Chaoyang Hospital (approval number: 2021-S-636).

### 2.2. Clinical data collection and follow-up

Upon admission to the emergency department, personal information, including age, sex, and history, as well as vital signs, such as body temperature, respiratory rate, heart rate, and blood pressure, were recorded. Furthermore, routine laboratory tests were conducted to determine white blood cell count, hemoglobin, hematocrit, platelet count, creatinine, albumin, CRP, and PC levels. Additionally, blood gas analysis was performed to measure pH, PaO_2_, PaCO_2_, and LAC levels. The G-CSF was collected from patients within the first 1 hour after admission to the emergency department. The levels of cytokines were determined using Human XL Cytokine Luminex® Performance Assay 46-plex Fixed Panel (Bio-Techne, United States). The SOFA and APACHE II scores were calculated based on the results of the first 24 hours of admission. All patients were followed up, and their survival statuses were recorded.

### 2.3. Statistical analysis

The statistical analysis was performed using SPSS 26.0 (IBM Corp, Armonk, NY) and Medcalc Statistical Software version 20.218 (Medcalc Software bvba, Ostend, Belgium). For quantitative data, the Student *t* test or the Mann–Whitney U test was applied depending on data distribution. Moreover, the qualitative variables were analyzed using the chi-square test. Quantitative data were expressed as mean ± standard deviation. Moreover, the median and interquartile ranges were used for the data that did not conform to the normal distribution. Logistic regression analyses were performed to evaluate the risk factors for 28-day mortality of sensitivity and specificity to determine the optimal cutoff value for differentiation. Eventually, area under the receiver operating characteristic curve (AUROC) comparisons were performed using the *Z* test with the MEDCALC software. A *P*-value < .05 was considered statistically significant.

## 3. Results

### 3.1. Baseline data of patients

The study included a total of 171 patients with sepsis. Among them, 122 patients (71.3%) belonged to the non-survivor group, while the remaining 49 patients (28.7%) were associated with the survivor group. The characteristics of patients are summarized in Table 1. There were no significant differences in gender, age, or comorbidities, including diabetes, hypertension, coronary heart disease, chronic obstructive lung disease, chronic renal failure, and cerebral vascular disease between the 2 groups *(P* > .05). The non-survivor group exhibited statistically significant higher scores for SOFA and APACHE II compared to the survivor group (*P* < .05). The non-survivor group showed a statistically significant higher LAC (1.3 (1.0, 1.9) vs 1.1 (0.9, 1.4)) and G-CSF (42.3 (19.7, 136.0) vs 15.7 (8.9, 22.8)) than the survivor group (*P* < .05). However, the systolic blood pressure (SBP), mean arterial pressure, and albumin in the non-survivor group were statistically lower than in the survivor group (*P* < .05).

**Table 1:** Comparison of patient baseline data.

	Survivor group (n = 49)	Non-survivor group (n = 122)	*P*
Gender (male/female)	31/18	67/55	.318
Age	71.0 (64.0, 83.0)	76 (65.0, 83.0)	.539
Comorbidities			
Diabetes (yes/no)	20/29	38/84	.227
Hypertension (yes/no)	22/27	54/68	.940
Coronary heart disease (yes/no)	16/33	28/94	.189
COPD (yes/no)	4/45	12/110	.734
Chronic renal failure (yes/no)	2/47	6/116	.815
Cerebral vascular disease (yes/no)	14/35	28/94	.440
SOFA	5.0 (3.0, 6.0)	9.0 (6.0, 10.3)	<.001
APACHE II	16.0 (12.0, 18.5)	22.0 (17.0, 28.0)	<.001
SBP	145.0 (130.5, 153.5)	131.5 (115.0, 143.0)	<.001
DBP	75.0 (65.0, 85.0)	72 (62.8, 81.0)	.192
MAP	96.0 (97.8, 105.8)	90 (81.0, 101.6)	.017
WBC (×10^9^/L)	8.0 (7.1, 12.1)	9.2 (7.1, 12.0)	.677
HGB (g/L)	124 (103.5, 137.5)	119 (105.0, 135.3)	.567
HCT (%)	37.9 (29.7, 43.6)	37.5 (32.4, 43.9)	.856
PLT (×10^9^/L)	200.0 (139.0, 283.0)	174.0 (135, 247.3)	.312
LAC (mmol/L)	1.1 (0.9, 1.4)	1.3 (1.0, 1.9)	.033
SCR (µmol/L)	72.5 (52.7, 85.4)	63.9 (46.5, 86.7)	.377
ALB (g/L)	36.3 (30, 39.4)	32.6 (24.5, 37.3)	.025
PCT (ng/mL)	0.05 (0.05, 1.12)	0.05 (0.05, 0.69)	.798
CRP (mg/L)	23.0 (8, 90.0)	14.5 (8.0, 81.0)	.466
G-CSF (pg/mL)	15.7 (8.9, 22.8)	42.3 (19.7, 136.0)	<.001

COPD = chronic obstructive pulmonary disease, SOFA = sequential organ failure assessment score, APACHE II = acute physiology and chronic health evaluation II, SBP = systolic blood pressure, DBP = diastolic blood pressure, MAP = mean arterial pressure, LAC = lactate, SCR = serum creatinine, ALB = albumin, PCT = procalcitonin, CRP = C-reactive protein, G-CSF = granulocyte colony-stimulating factor.

### 3.2. The predictive value of G-CSF, SOFA, APACHE II, and SBP for 28-day mortality in patients with sepsis

**Figure 1.**
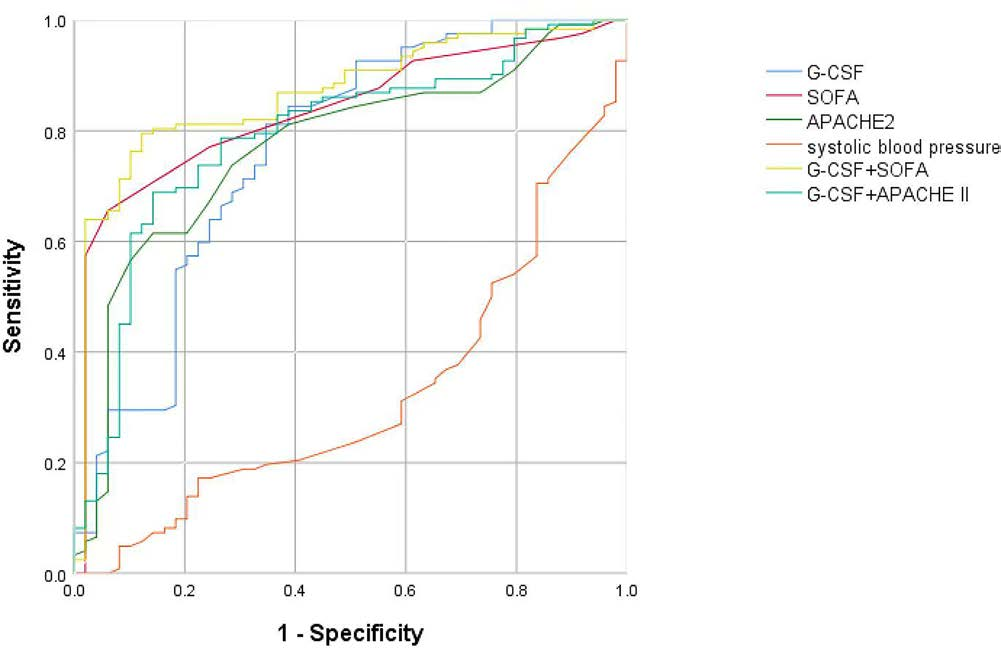
ROC curve of G-CSF, SOFA, APACHE II, and SBP predicting 28-day prognosis in patients with sepsis.

The results of multivariate regression analysis presented in Table 2 indicate that G-CSF, SOFA, APACHE II, and SBP are independent risk factors for 28-day mortality among patients with sepsis. The findings of this study indicate that there is a positive correlation between the increasing of G-CSF, SOFA, and APACHE II levels and the reduction in systolic blood pressure, which is accompanied by an increasing trend in the 28-day mortality rate of patients. Moreover, a receiver operating characteristic (ROC) curve analysis was performed to evaluate the predictive value of G-CSF. Our findings indicated that the G-CSF, SOFA, and APACHE II could predict the 28-day prognosis of patients with sepsis (Table 3 and Fig. 1). The AUROC of G-CSF was determined to be 0.765 with a cutoff value of 17.7, exhibiting a sensitivity of 81.1% and a specificity of 65.3%. This value was slightly lower than the AUROCs of SOFA (area under the curve [AUC] 0.845, cutoff 7.5, sensitivity 59.8%, specificity 95.9%) and APACHE II (AUC 0.771, cutoff 20.5, sensitivity 61.5%, specificity 85.7%). However, G-CSF demonstrated a higher sensitivity than the other 2 models.

**Table 2:** Results of multivariate regression analysis of G-CSF, SOFA, APACHE II, and SBP.

	B	SE	Wald	*P*	EXP (B) (95% CI)
G-CSF	0.002	0.001	5.659	.017	1.002 (1.000, 1.004)
SOFA	0.451	0.104	18.975	<.001	1.570 (1.282, 1.923)
APACHE II	0.139	0.040	12.004	.001	1.149 (1.062, 1.242)
SBP	‐0.036	0.011	11.841	.001	0.964 (0.945, 0.984)

G-CSF = granulocyte colony-stimulating factor, SOFA = sequential organ failure assessment score, APACHE II = acute physiology and chronic health evaluation II, SBP = systolic blood pressure, SE = standard error.

**Table 3:** Predictive value of G-CSF, SOFA, APACHE II, SBP, and 28-day prognosis in patients with sepsis.

	AUC	95% CI	*P*	Cutoff	Sensitivity (%)	Specificity (%)
G-CSF	0.765	0.680, 0.851	<.001	17.7	81.1	65.3
SOFA	0.845	0.782, 0.908	<.001	7.5	59.8	95.9
APACHE II	0.771	0.694, 0.848	<.001	20.0	61.5	85.7
G-CSF + SOFA	0.874	0.817, 0.931	<.001		73.8	89.8
G-CSF + APACHE II	0.791	0.716, 0.867	<.001		68.9	85.7
SBP	0.325	0.238, 0.412	<.001	142	73.0	59.2

G-CSF = granulocyte colony-stimulating factor, SOFA = sequential organ failure assessment score, APACHE II = acute physiology and chronic health evaluation II, SBP = systolic blood pressure, AUC = area under ROC curve, cutoff = the cutoff value was determined using the Youden method.

Furthermore, the *Z* test analysis showed nonsignificant differences in the ROC curves of G-CSF, SOFA, and APACHE II concerning the 28-day mortality rate among patients with sepsis (*Z*_1_ = 1.381, *P* = .167; *Z*_2_ = 0.095, *P* = .924). Furthermore, the combination of G-CSF with SOFA resulted in a superior predictive value for sepsis patients at 28-day (AUC 0.874, sensitivity 73.8%, specificity 89.8%, *Z* = 2.186, *P* = .029) compared to G-CSF alone. However, the predictive value of the combination of G-CSF and APACHE II did not exhibit a significant difference (AUC 0.771, sensitivity 61.5%, specificity 85.7%, *Z* = 0.494, *P* = .621) when compared to the use of G-CSF alone.

## 4. Discussion

Typically, patients who are acutely and critically ill are initially directed to the emergency department. Most of these critically ill patients are elderly patients with sepsis, heart disease, cerebrovascular disease, and kidney failure. Of these, sepsis remains an ongoing challenge, primarily because of the high mortality rate despite the provision of the best care.^[12]^ This presents a significant challenge for physicians as the treatment and prognosis for these patients are complicated. Consequently, accurate prognostication of mortality can assist medical practitioners in administering the most effective interventions to minimize hospitalization duration and associated expenses. Until now, several scoring systems have been externally validated to assess the severity of organ dysfunction, and various prognostic indicators have been proposed,^[2–5]^ including IL-6, tumor stimulating factor (TNF)-α, interferon-γ (IFN-γ), IL-7, and IL-12.^[13–15]^ And these cytokines demonstrate excellent diagnostic ability in sepsis patients. Furthermore, a novel clinical staging framework has been put forth for sepsis, wherein the identification of biomarkers that hold pivotal significance in the pathogenesis of sepsis is of greatest significance. The significance of biomarkers in sepsis diagnosis is progressively gaining prominence.^[16]^ Consequently, the objective of our study was to investigate the association between G-CSF and outcomes in patients with sepsis.

G-CSF is a glycoprotein, growth factor, and cytokine produced by a number of different tissues to stimulate the bone marrow to produce granulocytes and stem cells. It is widely used to reduce the duration of febrile neutropenia following cytotoxic chemotherapy^[17]^ and lymphopenia in patients with coronavirus disease 2019.^[18]^ Several previous studies have demonstrated that G-CSF could also be used as an infectious biomarker. Kawakami et al first proposed that G-CSF levels in adult patients with infection are significantly higher than in patients without infection.^[19]^ Moreover, there was no significant difference in G-CSF levels between patients with respiratory tract infections and those with urinary tract infections. However, the number of cases was insufficient, with only 56 cases, and no comparison has been made with acute viral infections or atypical pneumonia. Subsequently, Pauksen et al conducted a study comparing G-CSF levels in bacterial, viral, and atypical pneumonia infections and showed that G-CSF was rapidly increased in the blood in acute bacterial infections. However, there was no significant increase in G-CSF levels during acute viral infections or those caused by Mycoplasma pneumonia. Additionally, CRP was highly elevated in atypical pneumonia compared to viral infections. Therefore, they concluded that the measurement of G-CSF in the blood did not offer any clinical advantages over other well-established variables, including CRP in terms of the diagnostic distinction between viral and bacterial infections.^[20]^ Conversely, Zhang et al found that G-CSF was significantly increased in patients with influenza-associated pneumonia. Moreover, the combination of IL-6 and G-CSF biomarkers can enhance the accuracy of prognostic predictions for influenza-related pneumonia.^[21]^ And the role of G-CSF in the early diagnosis of fungal infections was poor compared to CRP, procalcitonin, IFN-γ, TNF-α, MIP-1β, IL-6, IL-8, IL-10, IL-12p70, and IL-17.^[22]^ Additionally, Li et al established mouse models infected with *Staphylococcus aureus* (*S aureus*) and *Klebsiella pneumoniae* (*K pneumoniae*), in which 6 cytokines, including IL-1β, IL-6, IL-12p70, G-CSF, IFN-γ, and TNF-α were significantly different (*P* < .05) between 2 bacterial infected groups. Regarding clinical samples, there were significant differences in the levels of 3 cytokines, including IL-6, IL-12p70, and G-CSF, between the infection groups (*S aureus* and *K pneumonia* group) and the negative control group. However, G-CSF was the only cytokine that exhibited the ability to distinguish between the negative control group, the group infected with *S aureus*, and the group infected with *K pneumonia*. Additionally, based on the ROC curves utilized to differentiate between the infected and noninfected groups, it was observed that the AUROC of G-CSF was the highest among the 6 cytokines (AUC = 0.9051). This finding was superior to that of IL-6 (AUC = 0.8227).^[23]^ Therefore, it is hypothesized suggest that G-CSF may serve as a potential biomarker for the early diagnosis of bloodstream infections and as a possible distinguishing feature between bloodstream bacterial infection caused by *S aureus* and *K pneumonia*. The determination of G-CSF levels may help in excluding life-threatening sepsis. The research results on the diagnostic value of G-CSF in local infections, such as ventilator-associated pneumonia, are completely opposite.^[24,25]^ Therefore, the application of G-CSF has great prospects, and further research is needed on G-CSF. For this purpose, our study demonstrated that the serum level of G-CSF was significantly higher (42.3 vs 15.7 pg/mL) in the non-survivor group than in the survivor group of patients with sepsis. The logistic analysis also showed that G-CSF can be an independent risk factor associated with 28-day mortality in sepsis patients, which helps with early judgment as well as treatment for emergency physicians.

The utilization of validated assessment tools such as the SOFA and APACHE II scores to monitor changes in patient status over time has been established in previous research as an efficient way of predicting mortality. As a consequence, these tools have gained widespread adoption in clinical practice.^[26–28]^ SOFA and APACHE II scores were analyzed and compared with G-CSF in terms of their prognostic value for 28-day mortality in patients with sepsis in our study. Herein, the logistic analysis revealed that SOFA and APACHE II scores were identified as independent risk factors for the prognosis of patients with sepsis. These findings are consistent with the above researches. Furthermore, the SOFA model exhibits superior predictive capacity, as evidenced by its highest AUROC value (AUC = 0.845) compared to the AUROCs of G-CSF and APACHE II, which were 0.765 and 0.771, respectively. Although SOFA and APACHE II had better specificity (95.9%, 85.7%), their sensitivities were poor (59.8%, 61.5%). Further *Z*-tests were conducted on SOFA, G-CSF, and APACHE II and showed that there were no significant differences among them for 28-day mortality in sepsis patients (*Z*_1_ = 1.381, *P* = .167; *Z*_2_ = 0.095, *P* = .924). However, when the cutoff value of G-CSF was 17.7 pg/mL, the sensitivity (81.1%) of prognosis was higher than the SOFA score.

The utilization of biomarkers alone for outcome prediction lacks complete accuracy. Therefore, a proposed approach involves the combination of biomarkers with severity scores to enhance predictive outcomes.^[29,30]^ Subsequently, we combined the biomarker G-CSF with SOFA and G-CSF together with APACHE II and found that G-CSF + SOFA and G-CSF + APACHE II showed better discrimination (AUC1 = 0.874, sensitivity 73.8%, and specificity 89.8%; AUC2 = 0.791, sensitivity 68.9%, and specificity 85.7%) for predicting the prognosis of patients with sepsis than G-CSF alone. However, the results indicate that only the combination of G-CSF and SOFA showed a statistically significant difference (*Z* = 2.186, *P* = .029). The combination of G-CSF and SOFA resulted in a sensitivity of 73.8%. And this combination had an outstanding advantage in clinical, considering that both G-CSF and SOFA score are very convenient to obtain.

The present study is subject to several limitations. First, the inherent bias of the single-center study design could not be avoided, so large sample size and multicenter studies are needed to verify the results. Furthermore, only a 1-time point (<1 hour after admission) was used to measure cytokines. Although early evaluations are useful for early prognostic information, the continuous variations of G-CSF may provide more insights. Third, some patients had received antiviral or antibiotic therapy before admission, and the effect of treatment on cytokines cannot be rule out.

## 5. Conclusion

The results of this study suggest that G-CSF, SOFA, APACHE II, and SBP are independent predictors of mortality among patients with sepsis. Moreover, the combined use of G-CSF and SOFA exhibits significant prognostic capability for mortality within 28 days in patients with sepsis.

## Author contributions

**Conceptualization:** Yugeng Liu, Bing Wei.

**Data curation:** Xiaomeng Fu, Ye Zhang.

**Investigation:** Xiaomeng Fu.

**Methodology:** Xiaomeng Fu.

**Resources:** Yugeng Liu.

**Supervision:** Junyu Wang.

**Writing – original draft:** Xiaomeng Fu.

**Writing – review & editing:** Yugeng Liu, Bing Wei.
